# Transcriptome Profiling Reveals Differential Gene Expression of Laccase Genes in *Aspergillus terreus* KC462061 during Biodegradation of Crude Oil

**DOI:** 10.3390/biology11040564

**Published:** 2022-04-07

**Authors:** Nada K. Alharbi, Mayasar I. Alzaban, Fawziah M. Albarakaty, Abeer R. M. Abd El-Aziz, Ahlam H. AlRokban, Mohamed A. Mahmoud

**Affiliations:** 1 Department of Biology, College of Science, Princess Nourah bint Abdulrahman University, P.O. Box 84428, Riyadh 11671, Saudi Arabia; nkalharbi@pnu.edu.sa (N.K.A.); mialzaban@pnu.edu.sa (M.I.A.); ahalrokban@pnu.edu.sa (A.H.A.); 2 Department of Biology, Faculty of Applied Science, Umm Al-Qura University, P.O. Box 715, Makkah Al Mukarramah 21955, Saudi Arabia; fmbarakati@uqu.edu.sa; 3 Botany and Microbiology Department, College of Science, King Saud University, Riyadh 11451, Saudi Arabia; 4 Molecular Markers Laboratory, Plant Pathology Research Institute, Agricultural Research Center, Giza 12619, Egypt

**Keywords:** *Aspergillus terreus* KC462061, biodegradation, qPCR, laccase, differential regulation, promoter

## Abstract

**Simple Summary:**

The Kingdom of Fungi is one of the most significant microorganism kingdoms, especially for soil fungi, which are still unexplored. Soil fungi play an extremely crucial role in the biodegradation of pollutants, mainly hydrocarbons. In this paper, molecular analysis delivers insights into laccase production by *Aspergillus terreus* KC462061 in the existence of crude oil, which is supported by the presence of five inducers, including aromatic compounds and metal ions. This paper established that the laccase of *A. terreus* KC462061 plays an essential function in the biodegradation of crude oil, and the synergistic effect of the Cu-ABTS compound caused an increase in laccase yields up to 22-fold after 10 days. This study confirmed that gas chromatography–mass spectrometry was a very accurate tool to demonstrate the biodegradation efficiency of *A. terreus* KC462061 for crude oil. The synergistic effect of the Cu-ABTS compound has the highest induction level of the transcription profile. *Lcc11* and *12* were the main *Lcc* genes in transcription profiles throughout the life cycle of *A. terreus* KC462061, and their transcript abundance was correlated with the Cu-ABTS compound. A quantitative real-time polymerase chain reaction (qPCR) was used for the analysis of the transcription profile of eight laccase genes in *A. terreus* KC462061. Cu-ABTS was highly effective for efficient laccase expression profiling, mainly via *Lcc11* and *12* transcription induction.

**Abstract:**

Fungal laccases have high catalytic efficiency and are utilized for the removal of crude oil because they oxidize various aliphatic and aromatic hydrocarbons and convert them into harmless compounds or less toxic compounds, thus accelerating the biodegradation potential of crude oil. Laccases are important gene families and the function of laccases genes varied widely based on transcription and function. Biodegradation of crude oil using *Aspergillus terreus* KC462061 was studied in the current study beside the transcription level of eight laccase (*Lcc*) genes have participated in biodegradation in the presence of aromatic compounds, and metal ions. Time-course profiles of laccase activity in the presence of crude oil indicated that the five inducers individual or combined have a very positive on laccase activity. In the status of the existence of crude oil, the synergistic effect of Cu-ABTS compound caused an increase in laccase yields up to 22-fold after 10 days than control. The biodegradation efficiencies of *A. terreus* KC462061 for aliphatic and aromatic hydrocarbons of crude oil were 82.1 ± 0.2% and 77.4 ± 0.6%, respectively. The crude oil biodegradation efficiency was improved by the supplemented Cu-ABTS compound in *A. terreus* KC462061. Gas chromatography–mass spectrometry was a very accurate tool to demonstrate the biodegradation efficiencies of *A. terreus* KC462061 for crude oil. Significant differences were observed in the SDS-PAGE of *A. terreus* KC462061 band intensities of laccase proteins after the addition of five inducers, but the Cu-ABTS compound highly affects very particular laccase electrophoresis. Quantitative real-time polymerase chain reaction (qPCR) was used for the analysis of transcription profile of eight laccase genes in *A. terreus* KC462061 with a verified reference gene. Cu^2+^ ions and Cu-ABTS were highly effective for efficient laccase expression profiling, mainly via *Lcc11* and *12* transcription induction. The current study will explain the theoretical foundation for laccase transcription in *A. terreus* KC462061, paving the road for commercialization and usage.

## 1. Introduction

Crude oil hydrocarbons are the most widespread environmental pollutants, including aliphatic and aromatic hydrocarbons (PAHs), which have been considered as serious ecological and public health concerns [1]. Traditional physical and chemical methods are highly complex and are expensive, and degradation is mainly based on crude oil composition, soil temperature, amount of oil contaminants, and these traditional methods are not effective to eliminate contaminants completely from the environment. Hence, biodegradation is a biological strategy that relies on the metabolic possibility of microorganisms to remove pollutants from the environment. Microorganisms use contaminants as the sole source of carbon for growth and metabolism. This strategy has been reported as the novel method for the removal of hydrocarbon contaminants. The use of the biological method has several advantages, including low costs, simple method and low energy consumption, and this method is suitable for the degradation of crude oil [2,3,4,5]. Several investigations reported the catabolic capacities of indigenous microorganisms such as algae [6], bacteria [7], and fungi [8] in the biodegradation of crude oil.

Some fungi have adapted to the polluted environments by particular enzyme systems that encourage them to utilize hydrocarbons as a sole carbon source. Different hydrocarbon-degrading fungi have been found in hydrocarbon-contaminated environments [9,10]. Numerous ascomycetes fungi produce high redox potential enzymes such as laccases (Lcc), lignin peroxidases (LiP), and manganese peroxidase (MnP), for the oxidation of lignin. These enzymes are capable of oxidizing a broad range of substrates, including hydrocarbons, insecticides, and plastics [8,11]. Among ascomycetes fungi [12], several Aspergillus species were promising in laccase production, *A. flavus* [13], *A. fumigates* [14], *A. nidulans* [15], *A. niger* [16], and *A. terreus* [17]. Wu et al. [18] have been used Lcc to remove PAH from the contaminated soil samples. The findings displayed that Lcc could completely degrade more than 15 PAHs within 24 h. Kucharzyk et al. [19] have been revealed the potentials of Lcc and manganese peroxidase from the indigenous bacteria isolated from the contaminated environment for the removal of PHAs. The biodegradation of PAHs was rapid and reached 80%. It is very useful to study the regulation of laccase transcription, which may be very beneficial for increasing the productivity of native laccases in fungi. Transcription level depends on environmental factors such as environmental pollutants (aliphatic and aromatic hydrocarbons) and available nutrient materials during the fungal life cycle [20,21].

Real time quantitative PCR (qPCR) is a very significant method in expression quantification because it is accurate, rapid, sensitive, and inexpensive. Recently, transcription of the *Lcc* genes family in *Cyathus bulleri* and *Pleurotus ostreatus* has been profiled in response to agricultural residues of polycyclic and aromatic hydrocarbons [22,23]. However, when it comes to *Aspergillus* species, there are few qPCR publications on the known *Lcc* gene families, despite there being very promising data in a transcript of *Lcc* genes due to isolate- and gene-specific factors [16,24]. 

The present study aimed to investigate the employment of *Aspergillus terreus* KC462061 on biodegradation of crude oil and estimate the transcription level of eight *Lcc* genes that participated in biodegradation in response to aromatic compounds and metal ions. The current study will lay the theoretical platform for unraveling the complicated molecular mechanism underpinning the expression of laccase and regulation in *A. terreus* KC462061, paving the road for commercialization and usage.

## 2. Materials and Methods

### 2.1. Fungal Isolate, Culturing Conditions, and Sample Preparation

*A. terreus* KC462061 [17] was cultured using potato dextrose agar medium (PDA) (Difco, Visalia, CA, USA) at 22 °C for seven days and the plates were stored at 2–8 °C. It was sub-cultured for every 15 days and the viability was tested. After seven days of incubation, mycelia plugs were cut from the PDA plate and inoculated into an Erlenmeyer flask containing 100 mL (Wertheim, Baden-Württemberg, Germany) of potato dextrose broth (PDB). The culture flasks were incubated for seven days at 28 °C in an orbital shaker incubator at 180 rpm. The preculture were completely homogenized using a glass homogenizer and centrifuged at 5000 rpm for 5 min. The fungal biomass was washed several times with double-distilled water and further inoculated in MSM medium without any crude oil (group 1) and the other group incorporated with 1% crude oil (group 2). For enzyme analysis, the two groups were incubated at 28 °C for 10 days. The samples were measured on the 3rd, 7th, and 10th days of treatment [25]. One mL of culture was centrifuged at 10,000 rpm for 5 min, and the culture supernatant was used as the crude sample for the determination of enzyme activity [26]. To analyze the impacts of different inducers on laccase production, ABTS (2,2′-azinobis (3-ethylbenzothiazoline-6-sulfonic acid), ferulic acid and guaiacol and two ions, Zn^2+^ and Cu^2+^ (zinc sulphate and copper sulphate), were used. The aromatic compounds and ions were added individually and combined to a concentration of 0.5 mM, and also MSM-supplemented by combining various inducers ions and aromatic compounds. A flask of the fungal isolate without any inducer was used as the control. Fresh MSM consisted of KH2PO4 (0.4 g/L), K2HPO_4_.3H_2_O (1.0 g/L), MgSO_4_.7H_2_O (0.025 g/L), NaNO_3_ (0.2 g/L), (NH4)_2_SO_4_ (0.1 g/L), and NaCl (0.5 g/L), with pH 7.0.

### 2.2. Lcc Activity Assay

The culture supernatant was used for the determination of Lcc activity. About 0.1 mL sample was mixed with 2.0 mL of 10 mM (2,2-azinobis (3-ethylbenzothiazoline-6-sulfonate (ABTS) prepared in sodium acetate buffer (0.1 M; pH 4.5). It was incubated for 30 min and the oxidation of ABTS was determined by measuring the absorbance of the sample at 415 nm against reagent blank [27].

### 2.3. Crude Oil Biodegradation

Biodegradation potential of crude oil was performed in Erlenmeyer flask (250 mL) containing crude oil (1%) as the sole source of carbon and energy. The culture medium was sterilized, and filter-sterilized crude oil was inoculated. A total of four agar plugs (5 mm diameter) of *A. terreus* KC462061 was taken from the PDA plate. It was aseptically transferred into the Erlenmeyer flask containing culture medium. All Erlenmeyer flasks were incubated for two weeks at 30 °C. 

#### 2.3.1. Extraction of Crude Oil

The residual crude oil from each sample was finally extracted using chloroform and an equal proportion of degradation medium. Anhydrous sodium sulfate was used to remove moisture from the produced crude oil. A rotary evaporator was used to evaporate the chloroform from the sample at 55 °C. The solvent was completely removed from the sample using a rotary evaporator under reduced pressure. The extracted residues were dissolved in minimum volume of chloroform (Sigma, New Brunswick, NJ, USA) and the amount was determined using a gas chromatography–mass spectrometry (GC-MS). 

#### 2.3.2. GC–MS Analysis of Crude Oil

Aliphatic and aromatic hydrocarbons fraction was analyzed by GC–MS equipped with a flame ionization detector. The temperature of inlet was maintained at 50 °C/min. The oven of GC-MS was held isothermally at 40 °C for 4 min and ramped 80 °C at 5 °C/min rate to 300 °C, and a final hold time of 20 min. Nitrogen was applied as carrier gas and the flow rate was 1.2 mL/min. The detector temperature was 300 °C. The flow rates of air and hydrogen gas were 400 and 40 mL/min, respectively. About 10 µL sample was injected and retention time was monitored for 35 min. The quantitative amounts of n-alkanes and aromatics were determined using GC–MS with n-C24D50 and DBT-D8 as internal standards, respectively [28]. All the experiments were performed in triplicate. The crude oil biodegradation efficiency was calculated using equation:

$$Crude\;oil\;biodegradation\;efficiency\;\left(\%\right)=\frac{C0-\mathrm{Ct}}{C0}\times 100$$

where C0 is the initial crude oil concentration and Ct is the crude oil concentration after 10 days of incubation. To get accurate data about aliphatic and aromatic hydrocarbons, we used mass spectra of these compounds Figures S1 and S2. 

### 2.4. Separation of A. terreus KC462061 Protein by SDS-PAGE

The protein profile of Lcc proteins in the culture, visualized by using 10% SDS-PAGE, was described previously [29]. The running and stacking gels contained acrylamide and bis-acrylamide with different concentrations. The samples were analyzed in vertical slab gel (thickness 1.5 mm × length 15 cm). Then, 25 µL of each sample’s protein extract was loaded into a polyacrylamide gel. The sample was run using a stable electric current (50 mA) at 28 ± 2 °C for 2.5 h. After the completion of protein separation, the protein bands were visualized using a silver staining method [30]. Standard protein ladder ranging between 66 and 22 kDa (Sigma-Aldrich, Darmstadt, Germany) for analysis and imaging gel by documentation system. Densitometric SDS-PAGE electrophoresis chromatogram available Figure S3. 

### 2.5. RNA Extraction and cDNA Synthesis

Mycelia from three samples (replicates) were harvested after 2, 4, 6, 10 and 12 days of incubation using an RNA extraction kit (Life Technologies, Pleasanton, CA, USA) based on manufactures instructions. The extracted RNA was quantified using a NanoDrop 2000 UV-Vis spectrophotometer (Thermo Scientific, Waltham, MA, USA). The purity of RNA was tested using agarose gel electrophoresis. The DNase treated RNA was converted as cDNA using a commercial kit (Bio-Rad, Hercules, CA, USA).

### 2.6. Specific Primers of Laccase Genes

qPCR primers for the eight *Lcc* genes (Lcc1-8) and GAPDH (glyceraldehyde-3-phosphate dehydrogenase) gene as housekeeping gene were designed by GeneLink (Orlando, FL, USA) (Table 1). The selected qPCR primers were specific and were evaluated using 1% agarose gel electrophoresis. The amplification potential of the selected primers was evaluated using the synthesized cDNA of each isoform tested at various dilutions (1:10). The calculated amplification efficiencies were 93.5–99.4% (R2 ≥ 0.98) and the size of the amplicon ranged between 102 and 199 bps for the *Lcc* encoding genes.

### 2.7. Transcription of Laccase Genes Using qRT-PCR

*Lcc* genes transcription profiles were evaluated under crude oil biodegradation in presence of five inducers in order to understand the nature of the relationship between the five inducers that impact on the transcription levels of different *Lcc* at various times during crude oil biodegradation. qPCR experiment was performed by mixing 5 µL SYBR GreenER qPCR SuperMix Universal, 1 µL primers (forward and reverse), template cDNA (2 µL) and Millipore double-distilled water. qRT-PCR was performed as described previously. The 2^−△△Ct^ relative quantification method [31,32] was utilized to calculate relative quantities of the laccase genes. Each reaction was performed in triplicate.

## 3. Results

### 3.1. Effects of Five Inducers on Laccase Production

**Figure 1:**
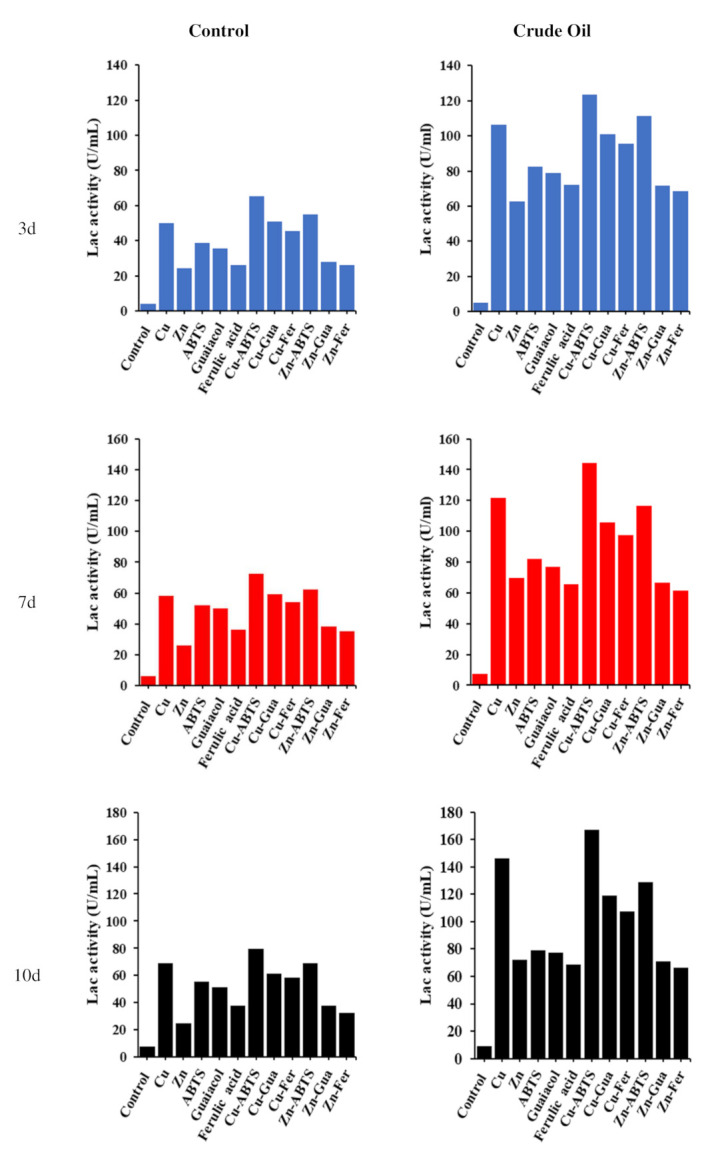
Time course of laccase activity monitored in MSM medium with five inducers (individual and combined) (control) and same medium, conditions 1% crude oil and same inducers (treatment).

In this study, it was exhibited that fungal isolate *A. terreus* KC462061 displayed the capacity to produce Lcc with or without crude oil supported by five inducers (Figure 1). Generally, *A. terreus* KC462061 showed Lcc enzyme activity in a medium without crude oil (control) but in a lower quantity during the 3rd, 7th, and 10th days. While the results of assaying at the same times showed that the enzyme activity of Lcc with crude oil had a higher quantity, which gradually increased during the experiment, the highest enzyme activity induction responses appeared after 10 days of incubation with crude oil. Time-course profiles of Lcc activity in the culture medium containing crude oil were highly indicated due to the five inducers, individual or combined, having a very positive effect on Lcc activity. In the case of the existence of crude oil, the addition of copper ions induces Lcc activity up to 15-fold (98.1 U/mL), whilst the synergistic effect of Cu-ABTS caused an increase in laccase levels up to 22-fold (130.4 U/mL) in comparison to the reference condition at 10th day (Figure 1).

### 3.2. Biodegradation of Aliphatic Hydrocarbons of Heavy Oil

**Figure 2:**
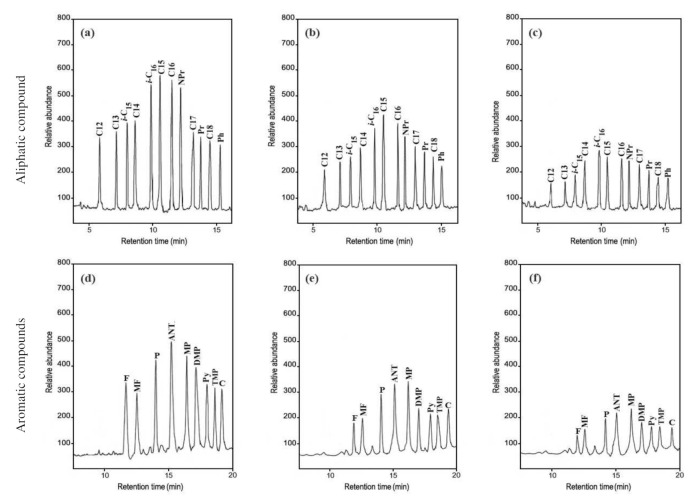
Gas chromatography–mass spectrometry (GC-MS) analysis of crude oil samples before and after biodegradation. (**a**) The aliphatic hydrocarbons of crude oil control; (**b**) biodegradation sample of *A. terreus* KC46206; (**c**) biodegradation sample of *A. terreus* KC46206 and Cu-ABTS. (**d**) The aromatic hydrocarbons of crude oil control; (**e**) biodegradation sample of *A. terreus* KC46206, (**f**) biodegradation sample of *A. terreus* KC46206 and Cu-ABTS, all treatments after 10 days.

The GC–MS analysis revealed that the *A. terreus* KC462061 supported by Cu-ABTS had a significant biodegradation potential on the n-Alkanes of the crude oil (Table 2 and Figure 2a–c). On day 10 of the biodegradation process by *A. terreus* KC462061 supported by Cu-ABTS, the biodegradation efficiency of the C12 to C20 increased rapidly to 80% (mean). However, for the *A. terreus* KC462061 biodegradation, the degradation potential of the C12 to C20 reached a maximum of 53.3% (mean). The Cu-ABTS supported *A. terreus* KC462061 by increased the biodegradation range and biodegradation rate of the n-Alkanes. The n-alkane availability in the crude oil effectively decreased mainly due to the biodegradation potential (Table 2), whereby the biodegradation efficiency range of *A. terreus* KC462061 supported by Cu-ABTS for C12 to C16 was 80.9 % to 83.1%. Compared with the *A. terreus* KC462061 only, the biodegradation efficiency of C12 to C16 was significantly increased, and the biodegradation efficiency improved in the range of 49.1% to 55.4%.

### 3.3. Biodegradation of the Aromatic Hydrocarbons of Crude Oil

The GC–MS analysis indicated that the presence of various PAHs in the crude oil effectively decreased due to the biodegradation potential (Table 3, Figure 2d–f). On day 10 of the biodegradation process by the *A*. *terreus* KC462061 induced by Cu-ABTS, the biodegradation efficiency of aromatic hydrocarbons was up to a maximum of 80.1%. Compared with the *A. terreus* KC462061 only, the biodegradation potential was reached up to 52.6%. In *A. terreus* KC462061, biodegradation efficiency was supported by Cu-ABTS for PAHs was 75.6% to 82.9%. In the case of used *A. terreus* KC46206, the biodegradation efficiency ranged from 48.7% to 58.8%.

### 3.4. SDS-PAGE of A. terreus KC462061 Protein Patterns

**Figure 3:**
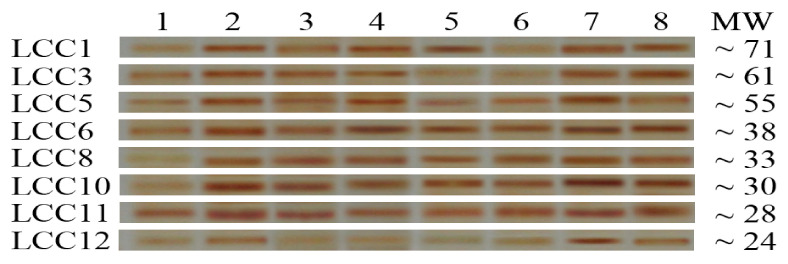
SDS-PAGE of Laccase profile of proteins found in the post-culture liquid of *A. terreus* KC46206 supplement with crude oil and different inducers after 10 days; 1, control; 2, addition Cu^2+^ to culture; 3, Zn^2+^; 4, ABTS, 5, guaiacol; 6, ferulic acid, 7, Cu_−_ ABTS and 8, Zn-ABTS. The protein molecular marker weights ranging from 66, 45, and 22 kDa was used.

The visualized electropherograms of Lcc bands in a culture of *A. terreus* KC462061 supplement with eight inducers and crude oil after 10 days were analyzed by SDS-PAGE (Figure 3). The band intensities of the individual eight Lcc, Lcc1, 3, 5, 6, 8, 10, 11, and 12 were noticed to have significant variations. In general, after treatment with eight inducers for fungal culture, these compounds were affected to develop a higher amount of putative Lcc bands, i.e., its overproduction. Cu-ABTS was the most effective inducer addition among all putative Lcc bands. Therefore, Cu-ABTS most likely impacts the translation level of individual Lcc.

### 3.5. RT-PCR

**Figure 4:**
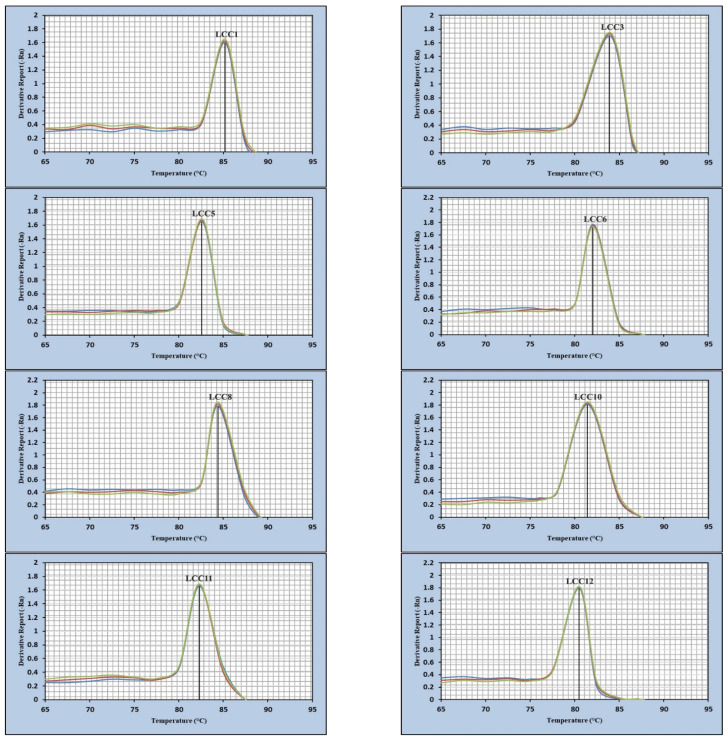
Melting curves of the *Lcc* gene amplicons of *A. terreus* KC46206.

Through the establishment of melting-curve-based monoplex real-time PCR, equivalent melting temperatures (Tm) of each primer pair was confirmed by detecting each gene in the RT-PCR analysis (Figure 4). The optimum selected primers useful to amplify the corresponding *Lcc* genes generating a dissociation curve with a specific peak and the Tm values of all amplicons were as follows: 85.17 °C for *Lcc1*, 83.97 °C for *Lcc3*, 82.64 °C for *Lcc5*, 84.41 °C for *Lcc8*, 81.45 °C for *Lcc10*, 82.31 °C for *Lcc11* and 80.51 °C for *Lcc12*.

### 3.6. Transcription Profiling of A. terreusKC46206 eight Laccase Genes

**Figure 5:**
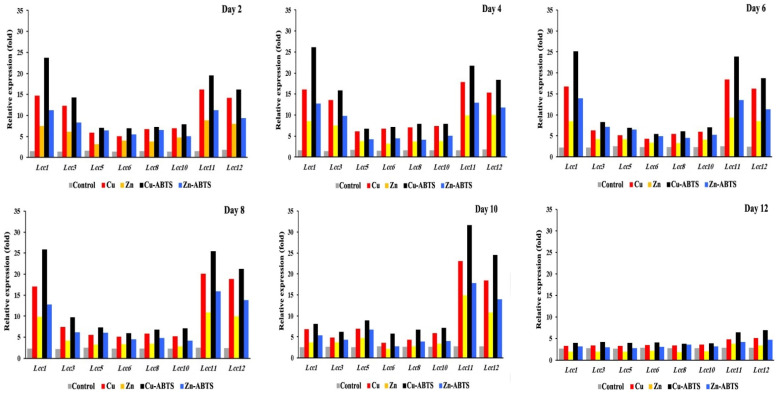
Transcription profile of laccase encoding genes during the growth of *A. terreus* KC46206 in the liquid cultures, supported by 1% crude oil at different growth times: 2nd day, 4th day, 6th day, 8th day, 10th day and 12th day. Fold-transcription is reported relative to the GAPDH gene of *A. terreus* KC46206.

The transcriptional patterns of the eight laccase genes, *Lcc1*, *Lcc3*, *Lcc5*, *Lcc6*, *Lcc8*, *Lcc10*, *Lcc11*, and *Lcc12*, were assessed on the second, 4th, 6th, 8th, 10th, and 12th days of fungal development, supported by Cu, Zn ions, Cu-ABTS, Zn-ABTS, and 1% crude oil, and the findings are given in Figure 5. All *Lcc* genes were demonstrated to be stimulated in the assayed conditions, with induction levels varying from two- to fifteen-fold higher than in the reference conditions.

**Figure 6:**
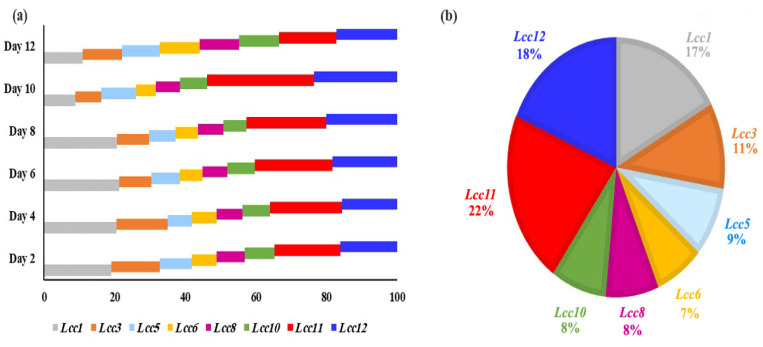
Relative transcript abundance (expressed as a percentage) of the *Lcc* genes. (**a**) Relative transcript abundance of the eight laccase genes in each day. (**b**) Relative transcript abundance of the eight laccase genes during all days. The total transcript level of all *Lcc* genes was taken as 100%.

*Lac1*, *3*, *8*, and *11* had the most significant changes in transcription levels (>10-fold). The other genes, *Lcc5*, *6*, *8*, and *10*, have low levels of significance on days 2 and 4. The genes have new levels of significance on days 6 and 8, where transcription levels of Lcc1 (11.1- and 11.5-fold, respectively), *Lcc11* (9.6- and 10.2-fold, respectively) and *Lcc12* (7.8- and 8.9-fold, respectively) based on induction by Cu-ABTS, comparing the corresponding expression levels on day 6 and 8. On day 10, the transcriptional profiles of *Lcc11* and *12* were the most highly significant during this stage. On day 12, the process of translation was very slightly down-regulated for all *Lcc* genes. Therefore, the gene expression levels were changed in all *Lcc* genes by up to two-fold or less, and four inducers failed to produce a powerful induction for all *Lcc* genes, so transcription has almost stopped at this stage. In general, Cu-ABTS have highest induction level of transcription process. Among the eight *Lcc* genes, *Lcc1*, *11* and *12* were predominantly expressed from 2 to 8 days, but on the 10th day, only *Lcc* 11 and 12 were predominantly expressed (Figure 6a). *Lcc1*, *11*, and *12* were the most abundantly expressed at 17%, 22%, and 18%, during transcription for 12 days (Figure 6a), but other *Lcc* genes transcription was negligible relative to abundant genes (Figure 6b).

## 4. Discussion

In this study, it was demonstrated that fungal isolate *A. terreus* KC462061 has the ability to produce Lcc with an inducer (crude oil) and without crude oil, supported by five inducers. The synergistic action of Cu-ABTS increased laccase levels up to 22-fold in the culture medium containing inducer, and in comparison to the reference condition. The production of Lcc produced by *Aspergillus* sp. HB_RZ4 over a 9-day period was measured as a response to eight significant variables, including CuSO_4_. Optimum yield (9.20 µ/mL) Lcc activity was observed under specific conditions, including CuSO_4_ (0.001 g/L) [33]. A similar trend, higher induction levels, was observed with the addition of 1mM copper sulfate, while laccase activity of *Aspergillus flavus* JF683612 developed from 5.1 µ/mL to 51.84 µ/mL(10-fold) [13]. *Pleurotus ostreatus* has been cultured using PDY medium in submerged culture containing copper sulphate (CuSO_4_) or both copper sulphate and ferulic acid (Cu–Fer). Supplementation of Cu^+2^ considerably increased laccase activity up to 10-fold, and Cu and ferulic acid (Cu-Fer) supplemented culture medium showed synergistic activity and improved Lcc levels up to 40-fold than the control [34]. Cu^2+^ ions were the most widely utilized inducer of Lcc production, and they were required for high Lcc yields in *Cerrena* sp. HYB07. Cu^2+^ and Zn^2+^ ions promoted Lcc activity by 99.95 and 31.78%, respectively. The highest induction reactions have occurred with guaiacol and ABTS, which enhanced laccase production by 26.7 and 40.1%, respectively, at 250 µM [21].

In the current study, the biodegradation efficiency of *A. terreus* KC462061, supported by Cu-ABTS for aliphatic and aromatic hydrocarbons, increased up to 83.1 and 80.1%, respectively. Many enzymes found in filamentous fungi have been shown to aid in hydrocarbon biodegradation and mineralization. For instance, biodegradation of crude oil was highly effective with high Lcc enzyme production from *A. terreus* KC462061 [35]. Fungal Lcc has been characterized from various genera, including *Penicillium* and *Curvularia* [36]. Fusarium sp. F092 has been showed improved biodegradation potential of aliphatic hydrocarbon in crude oil type-1 (49%), crude oil type-2 (72%), and crude oil type-3 (98%) after 20 days. Fusarium sp. F092 has been showed improved manganese peroxidase (69.2 U/mL) and Lcc (48.8 U/mL) after 20 days of culture [37]. On the 9th and 6th days, *Aspergillus oryzae* had the greatest Lcc activities of 36.0 and 27.37 µ/mL, respectively, whereas the 3rd day had the lowest Lcc activity of 2.11 µ/mL. *A. oryzae* had the best performance in biodegrading all of the hydrocarbons in used motor oil [10]. *Aspergillus niger* and *Aspergillus oryzae* have been demonstrated to improve crude-oil degradation in Bushnell Haas medium and achieved 54% and 99% oil degradation, respectively, in the optimized medium [38]. Prior studies on the aromatic biodegradation, particularly of PAHs by *Aspergillus fumigatus*, revealed considerable biodegradation of anthracene. After 5 days, the biodegradation efficiency could be kept at around 60% [39]. *Aspergillus terreus* has been characterized from polluted soil contaminated with polycyclic aromatic hydrocarbon and metabolized Pyrene and benzo(a)pyrene [40,41]. Benguenab and Chibani [42] confirmed the application of *Aspergillus ustus* on crude oil and diesel biodegradation, exhibited the highest level of degrading at 30.43% and 21.74%, respectively. *Trematophoma* sp. UTMC 5003 has been a very effective isolate and removed 70% of crude oil from the oil-polluted soils within 15 days of treatment, and Lcc activity increased over four-fold in the presence of the inducer (crude oil) [43]. Fungi produced the Lcc enzyme as secondary metabolites. When available, good carbon or nitrogen concentrations in the materials where the fungus grows may impact Lcc enzyme production and activity [20]. Lcc is one of the multi-copper phenol enzymes that has been well documented in several fungal species to serve varied functions in the oxidation of various aromatic and phenolic compounds using oxygen [44].

In present study, the band intensities of individual eight Lcc were noticed significant variations. In general, after treatment with eight inducers for fungal culture, these compounds were affected to develop a higher amount of putative Lcc bands, i.e., its overproduction. Cu-ABTS was the most effective inducer addition among all putative Lcc bands. Electrophoresis techniques provide an outstanding opportunity to analyze the substructure variation in proteins among different fungal isolates and can be a perfect biochemical marker at the fungal species levels [45]. SDS-PAGE was carried out to observe the change in gene expression of *A. terreus* treated with AgNPs and CuNPs. The results revealed the lowest percentage of polymorphism (16.67%), and the protein profile of Lcc displayed a total of six bands, which were recorded in *A. terreus*. Protein patterns included 78, 71, 61, 53, 48, and 37 kDa, which were induced by AgNPs and CuNPs [46]. PAGE gel of *A. flavus* distinctive bands were detected at 78, 61, 48, 28 60 kDa, and belong to the Lcc enzyme induced by copper ions. The increase in copper concentration added to the medium lead to the induction of the laccase enzyme, which is usually the fundamental mechanism responsible for the decolorization and degradation of a number of dyes [13]. The PAGE profile of laccase produced by Cerrena unicolor included six bands, Lcc1, 2, 3,4,5, and 6, with a range of the molecular masses from 75 to 40 kDa. The addition of Cu+2 or Mn+2 ions elicited Lcc bands after 48 h, up to 7- and 9-fold, respectively, as compared to the control conditions. The intensities of the putative Lcc bands varied, with Lcc3, 4, 5, and 6 bands showing greater overproduction as compared to Lcc1 and 2 [47]. 

Our results presented evidence of an accurate method, based on real-time PCR monitored with fluorescent cDNA binding dye and followed by melting curve analysis. The melting curve analysis was able to detect specific genes with high degrees of sensitivity and specificity and discriminate between them. Specific amplification of the genes Lcc1, 2, 3, 4, 5, 7, 8 of *Cerrena* sp. HYB07 were confirmed by a single peak in melting curve analysis [21].

In this study, it was found that Cu-Fer has a maximum increase in the expression of tested *Lcc* genes. We selected GAPDH as the reference gene because It is the most appropriate reference gene for the analysis of transcription profile in *Lcc* genes. In qPCR analysis, housekeeping genes have been widely used as normalization references, which may indicate correct expression with varied experimental conditions [48]. An evident link has been reported between biodegradation inducers and the expression of *Lcc* genes [49]. A total of 13 *Lcc* encoding genes have been identified from the whole genome of Cyathus bulleri. In this species, the transcripts for all Lcc genes considerably decreased as growth increased, except the *Lcc1*, *3*, *12* genes. This study proposed that the gene *Lcc12* was considered as the one of the major contributor to laccase transcription. Results indicated that *Lcc12* was mainly transcribed during the early and later stages of life cycle. The genomic similarities of all *Lcc* genes (excluding *Lcc12*) revealed that they evolved as a consequence of duplication of gene and were closely related with each other [22]. Biodegradation of polycyclic aromatic hydrocarbons (PAHs) using *Pleurotus ostreatus* could induce the expression levels of six *Lcc* genes. Each naphthalene and tween 80 individually could act as an inducer for the expression of *Lcc* genes. The combination of naphthalene and tween 80 resulted in the greatest promotion of the expression of various Lcc genes. The expression of *Lcc1* (37.4-fold), *Lcc2* (10.1-fold), *Lcc3* (30-fold), *Lcc4* (56.7-fold), *Lcc1* (70.6-fold) and *Lcc10* (67.8-fold) were considerably high; additionally, a higher concentration of inducer such as CuSO_4_ improved the expression of various *Lcc* genes, including *Lcc2* and *Lcc10* genes. *Lcc10* and *12* genes have been considered as the major contributors (highest expression level) in the case of *P. ostreatus* [23]. *Lcc7* gene was the predominant of all *Lcc* genes expressed in the culture of *Cerrena* sp., and its transcript quantity has been associated with *Lcc* inducers such as Cu, Zn, ABTS, and guaiacol. Lcc7 transcript levels increased 1000-fold when Cu^+2^ ions were incorporated with the culture medium. In the presence of Cu^2+^ ions in the culture medium, transcription level has been lower for *Lcc4* genes. Expression of *Lcc* genes such as *Lcc2*, *Lcc3*, *Lcc5*, *Lcc6*, and *Lcc8* has been considerably suppressed by presence of Zn^2+^ ions in the culture medium [21]. Multiple potential transcription regulatory locations were discovered inside the promoters of the *Lcc* genes, indicating that each *Lcc* gene was stimulated by one or more factors. For example, promoters of *Lcc1*, *4*, *7*, and *8* were expected to be metal response elements (MREs). *Lcc6* promoter contained a high level of angiotensin-converting enzyme 1 (ACE1). The promoters of genes *Lcc3* and *Lcc5* have not been associated with ACE1 or MRE sites. Xenobiotic response elements have been characterized in various promoters except for *Lcc6*, *2* and *1* promoters [21].

Four putative ACE1 and two MREs elements found in the promoter part of the Lcc gene of *Trametes velutina* have been shown to enhance *Lcc* gene expression by various metals, including Fe^2+^ and Cu^2+^. Additionally, the one XREs element that can be found in the promoting region of the *Lcc* gene may act as the potential regulatory element for transcription of genes by the induction of supplemented aromatic compounds in the medium. All putative ACE1, MREs, and XREs elements located in the promoter region of the Lcc genes may be extremely linked involved in the transcription of *Lcc* gene; nevertheless, the specific mechanism of action of relationship between these elements and the ability to induce transcription remain unclear [50]. The addition of copper sulphate and ferulic acid with the culture medium resulted in the significant induction of *Lcc2*, *9*, and *10*, as well as, to a lesser extent, for other *Lcc* genes, owing to the existence of only one MREs and XREs induction pathway for these genes or, more likely, due to the multiple pathways for MREs and XREs mapped in their promoter sites [34].

*Lcc7* of Trametes versicolor was robustly induced by Cu^2+^ ions, but not found to have ACE1 or MREs as activated transcription factors binding to the promoter site. *Lcc7* most likely included a non-conventional element or induced Cu^2+^ ions induction; however, this may be due to a mechanism other than ACE1 and MREs. There was no obvious causal link between an element’s presence and the usual reaction, or between the number of elements and the strength of the projected response [51]. It has been reported the variation of *Lcc* genes expression based on the environmental pollutants in the natural environment. In natural environment, the amount and the nature of the substrate varied widely and time dependent. Environmental pollutants lead to oxidative stress and these pollutants involved in the regulation of *Lcc* gene expression [52].

## 5. Conclusions

The present work delivered insights into laccase production by *A. terreus* KC462061 in the existence of crude oil. Two ions, Zn^2+^ and Cu^2+^, and three aromatic compounds, ABTS, ferulic acid and guaiacol, exerted a remarkable influence on its laccase yields, especially Cu-ABTS. Our results in this paper establish that Lcc of *A. terreus* KC462061 plays an essential function in fungus adaptation to harsh environmental conditions such as the biodegradation of crude oil. *A. terreus* KC462061 could degrade aliphatic and aromatic hydrocarbons in crude oil in the culture medium supplemented with Cu-ABTS. This study established the complete transcription profile of eight *Lcc* genes from *A. terreus* KC462061 during the biodegradation process of crude oil. The synergistic effect of Cu-ABTS compound has the highest induction level of transcription profile. *Lcc11* and *12* were the main *Lcc* genes in transcription profiles throughout the life cycle of *A. terreus* KC462061, and their transcript abundance was correlated with Cu-ABTS compound with a validated reference gene.

## Figures and Tables

**Table 1:** List of primers used for the qRT-PCR of *Lcc* genes in this study.

Genes	Primer Sequence		Amplicon Length (bp)
	Forward Primer (5′–3′)	Reverse Primer (5′–3′)	
*Lcc1*	ATGGTTGAGACCGATTTGCAC	AAGAAGGACGGGAACATTAGGG	159
*Lcc3*	TTGCGATCTTGCCTGTTCTC	CATCAGGTGCCAGAGCTTGA	199
*Lcc5*	GGCAGGAGGTGCTGACTACAA	ACGACCTTATTGCGGGCTAAC	195
*Lcc6*	GCAGCTTTACTCGCTATTCCA	TCAAAGCCATCAGGGCTAAC	104
*Lcc8*	TCAAATTCCACTTCCCCTACCA	GGACCAAAATAAGAGAGCCAGG	199
*Lcc10*	ATGGGGTCTACCAGAAGGGTA	TGGGAGTGATACCAATATGTGC	136
*Lcc11*	GGACAATGCATTCAACGAAG	AGAAAGTGCCGGTCTGGTC	107
*Lcc12*	GAAGTCCACGCCTATGATGAA	CAGGGTTGGCAATACTAACGA	103
*GAPDH*	GTTCAAGTACGATTCCGTCCA	TTCTCAGCGAAGACGGTGAC	102

**Table 2:** Biodegradation efficiencies of *A. terreus* KC462061 and *A. terreus* KC462061 + Cu-ABTS for C12 to C20 n-alkanes after 10 days.

n-Alkanes	*A. terreus* KC462061 Biodegradation			*A. terreus* KC462061 + Cu-ABTS Biodegradation		
	Initial Abundance *	Residual Abundance	Degradation Efficiency (%)	Initial Abundance	Residual Abundance	Degradation Efficiency (%)
C12	16.4 ± 0.46	7.3± 0.33	55.4 ± 0.10	14.2 ± 0.41	2.8 ± 0.09	83.1 ± 0.51
C13	15.6 ± 0.91	7.4 ± 0.78	52.5 ± 0.12	11.6 ± 0.37	2.1 ± 0.13	81.8 ± 0.14
i-C15	20.7 ± 0.73	9.4 ± 0.29	54.5 ± 0.48	15.1 ± 0.40	2.6 ± 0.45	82.7 ± 0.66
C14	22.8 ± 0.28	11.6 ± 0.23	49.1 ± 0.64	18.7 ± 0.82	3.4 ± 0.63	81.8 ± 0.28
i-C16	32.8 ± 0.69	14.9 ± 0.12	54.7 ± 0.31	25.6 ± 0.57	4.9 ± 0.98	80.9 ± 0.36
C15	36.9 ± 0.42	17.1 ± 0.28	53.6 ± 0.19	30.9 ± 0.14	5.9 ± 0.92	80.8 ± 0.47
C16	26.8 ± 0.76	13.2 ± 0.69	50.7 ± 0.89	21.3 ± 0.17	4.1 ± 0.84	80.2 ± 0.86
NPr (C18)	32.9 ± 0.15	14.3 ± 0.39	56.5 ± 0.56	26.4 ± 0.49	5.7 ± 0.61	78.4 ± 0.40
C17	35.4 ± 0.59	16.3 ± 0.17	53.9 ± 0.24	29.2 ± 0.13	6.7 ± 0.23	77.1 ± 0.58
Pr (C19)	23.9 ± 0.81	12.1 ± 0.33	49.3 ± 0.91	18.6 ± 0.64	4.1 ± 0.49	77.9 ± 0.38
C18	28.3 ± 0.71	15.3 ± 0.24	46.6 ± 0.59	22.1 ± 0.19	5.6 ± 0.02	74.6 ± 0.74
Ph (C20)	22.1 ± 0.29	10.7 ± 0.27	51.5 ± 0.36	16.3 ± 0.34	4.7 ± 0.71	71.1 ± 0.59

regular C15 = isoprenoid, regular C16 = isoprenoid, NPr = norpristane, Pr = Pristane, ph = phytane. * Data represent mean of three replicates ± Standard Deviation.

**Table 3:** Biodegradation efficiencies of *A. terreus* KC462061 and *A. terreus* KC462061 + Cu-ABTS for C13 to C18 PAHs after 10 days.

PAHs	*A. terreus* KC462061 Biodegradation			*A. terreus* KC462061 + Cu-ABTS Biodegradation		
	Initial Abundance *	Residual Abundance	Degradation Efficiency (%)	Initial Abundance	Residual Abundance	Degradation Efficiency (%)
F (C13)	5.1 ± 0.42	2.1 ± 0.17	58.8 ± 0.98	4.7 ± 0.13	0.9 ± 0.18	82.9 ± 0.47
MF (C14)	4.2 ± 0.75	1.9 ± 0.21	54.7 ± 0.52	3.9 ± 0.48	0.7 ± 0.56	82.1 ± 0.69
P (C14)	6.2 ± 0.62	2.9 ± 0.09	53.2 ± 0.73	5.7 ± 0.66	1.1 ± 0.58	80.7 ± 0.25
Anthracene (C14)	7.4 ± 0.85	3.4 ± 0.39	54.1 ± 0.26	6.7 ± 0.89	1.4 ± 0.31	79.1 ± 0.49
MP (C15)	5.7 ± 0.15	2.7 ± 0.11	52.6 ± 0.56	5.4 ± 0.55	1.2 ± 0.54	77.7 ± 0.58
DMP (C16)	5.3 ± 0.11	2.6 ± 0.26	50.9 ± 0.43	4.9 ± 0.42	1.1 ± 0.27	77.5 ± 0.89
Py (C16)	4.2 ± 0.59	2.1 ± 0.17	50.0 ± 0.17	3.9 ± 0.79	0.8 ± 0.11	79.4 ± 0.33
TMP (C17)	4.3 ± 0.71	2.1 ± 0.25	51.1 ± 0.15	3.9 ± 0.33	0.9 ± 0.09	76.9 ± 0.94
C (C18)	4.1 ± 0.36	2.1 ± 0.19	48.7 ± 0.90	3.7 ± 0.21	0.9 ± 0.09	75.6 ± 0.41

F = fluorene, MF = methylfluorenes, P = phenanthrene, MP = methylphenanthrenes, DMP = dimethylphenanthrenes, Py = pyrene, TMP = trimethylphenanthrenes, C= chrysene. * Data represent mean of three replicates ± Standard Deviation.

## Data Availability

Not applicable.

## Supplementary Materials

The following are available online at https://www.mdpi.com/article/10.3390/biology11040564/s1, Figure S1: Mass spectra of aliphatic hydrocarbons (a) isoprenoid (C16), (b) norpristane (C18), (c) Pristane (C19) and (d) phytane (C20), Figure S2: Mass spectra of aromatic hydrocarbons (a) methylphenanthrenes (C15), (b) pyrene (C16), (c) trimethylphenanthrenes (C17) and (d) chrysene (C18), Figure S3: Densitometric SDS-PAGE electrophoresis chromatogram of (a) control (A. terreus KC46206), (b) addition Cu2+ to fungal culture, (c) addition Zn2+, (d) addition ABTS, (e) addition guaiacol, (f) addition ferulic acid (g) addition Cu- ABTS, (h) addition Zn-ABTS.
