# Supplementary material for: Transcriptome Profiling Reveals Differential Gene Expression of Laccase Genes in Aspergillus terreus KC462061 during Biodegradation of Crude Oil
Source: Biology (Basel). 2022 Apr 7;11(4):564. doi: 10.3390/biology11040564 (PMC9026905; doi:10.3390/biology11040564)
Supplement: Supplementary file 1 [file biology-11-00564-s001.zip › biology-1649310-supplementary.pdf]

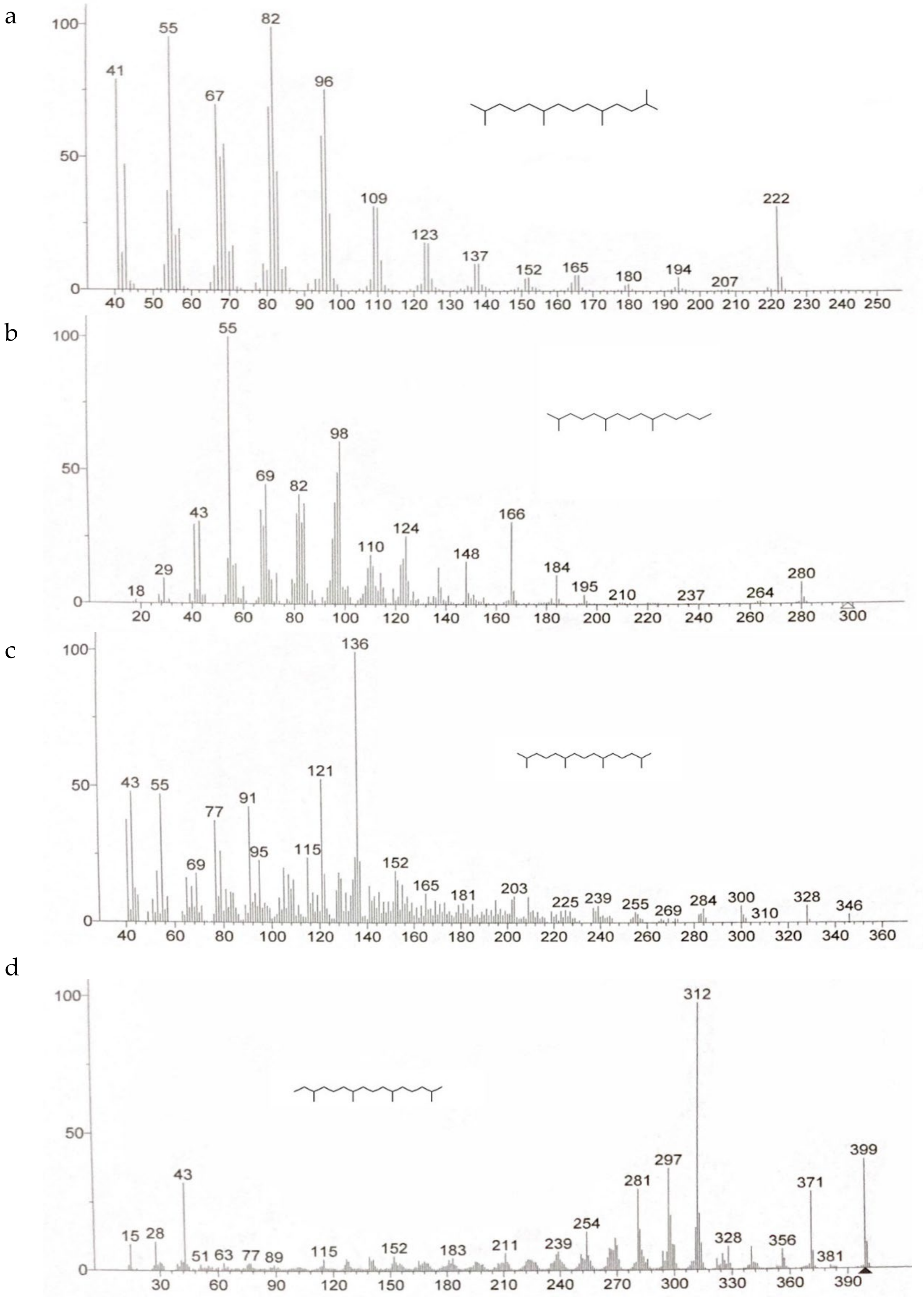

Figure S1: Mass spectra of aliphatic hydrocarbons (a) isoprenoid (C16), (b) norpristane (C18), (c) Pristane (C19) and (d) phytane (C20).

a

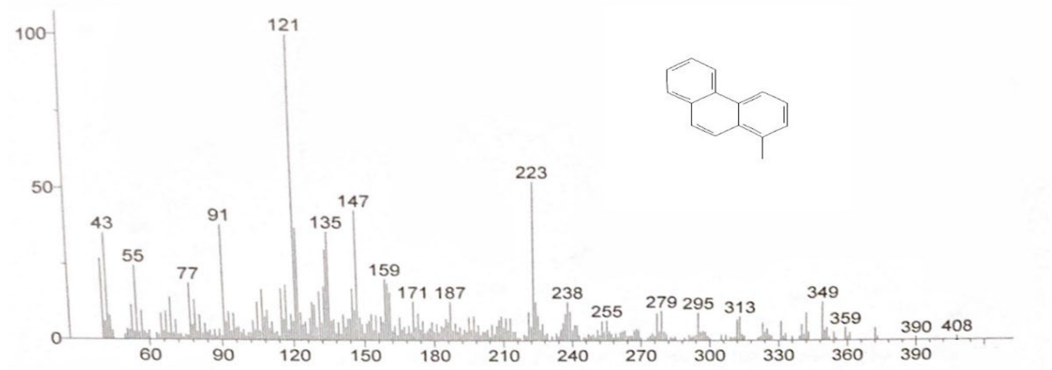

b

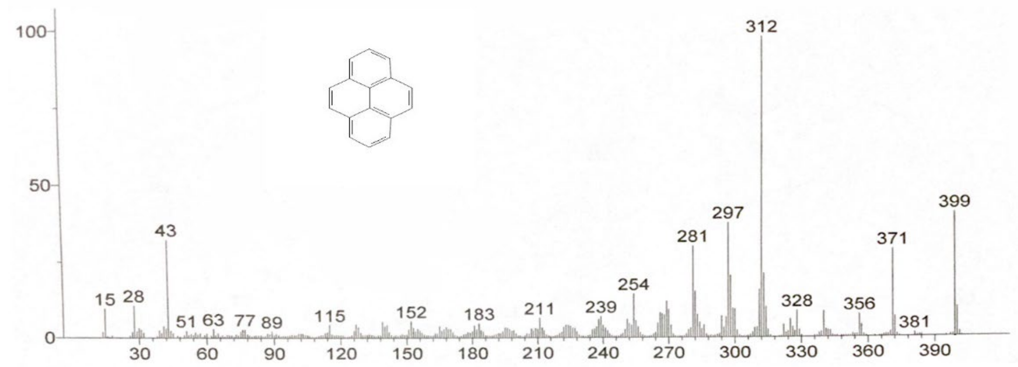

c

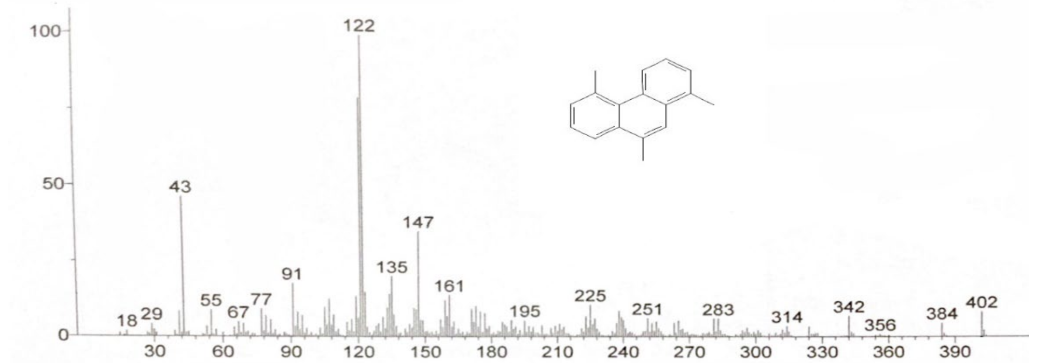

d

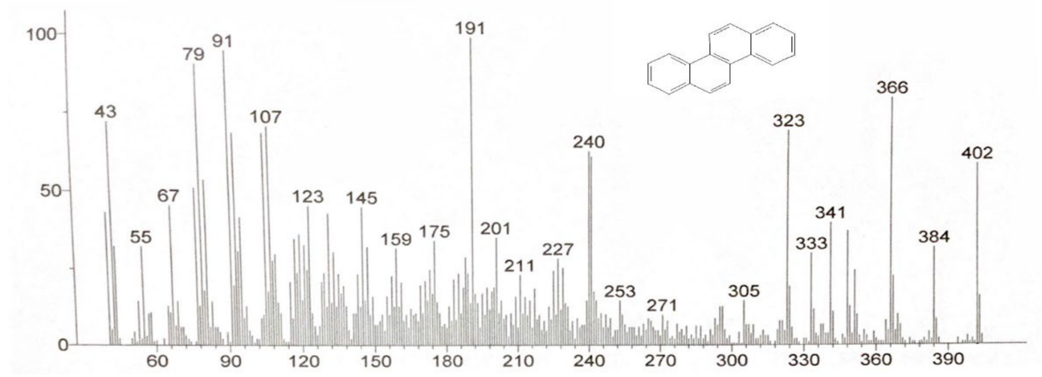

Figure S2: Mass spectra of aromatic hydrocarbons (a) methylphenanthrenes (C15), (b) pyrene (C16), (c) trimethylphenanthrenes (C17) and (d) chrysene (C18).

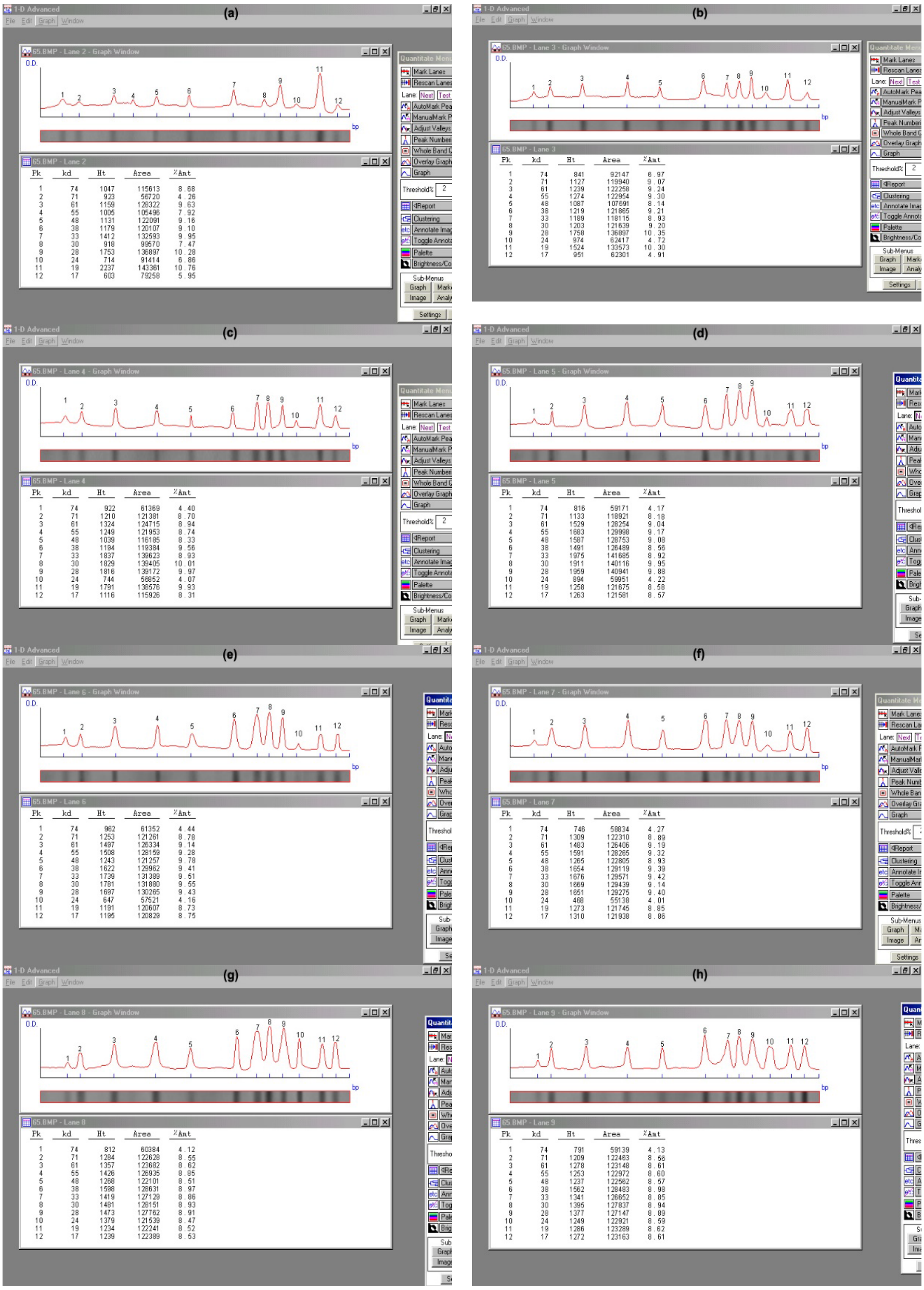

Figure S3: Densitometric SDS-PAGE electrophoresis chromatogram of (a) control (*A. terreus* KC46206), (b) addition Cu2+ to fungal culture, (c) addition Zn2+, (d) addition ABTS, (e) addition guaiacol, (f) addition ferulic acid (g) addition Cu- ABTS, (h) addition Zn-ABTS.
